# Regulación y prohibición del asbesto en América Latina y el Caribe: análisis comparativo de política

**DOI:** 10.15446/rsap.V27n2.118081

**Published:** 2025-03-01

**Authors:** Fabio A. Escobar-Díaz, Carlos A. Orozco-Castaño

**Affiliations:** 1 FE: Soc. M. Sc. Salud Pública. Ph. D. Salud Pública. Fundación Universitaria el Área Andina, Facultad de Ciencias de la Salud y del Deporte. Bogotá, Colombia. fescobarl3@areandina.edu.co Fundación Universitaria del Área Andina Fundación Universitaria el Área Andina Facultad de Ciencias de la Salud y del Deporte Bogotá Colombia fescobarl3@areandina.edu.co; 2 CO: Biol. M. Sc. Ciencias Biológicas, M. Sc. Ciencias Biomédicas. Ph. D. Ciencias Biomédicas. Instituto Nacional de Cancerología. Bogotá, Colombia. corozco@cancer.gov.co Instituto Nacional de Cancerología Bogotá Colombia corozco@cancer.gov.co

**Keywords:** Amianto, legislación, regulación gubernamental, política pública, América Latina *(fuente: DeCS, BIREME)*, Asbestos, legislation, government regulation, public policy, Latin America

## Abstract

**Introducción:**

El asbesto es un mineral empleado en diferentes actividades industriales, pero tiene un impacto significativo en la salud de la población trabajadora. Diversos países han establecido políticas para prohibir o regular su uso.

**Objetivo:**

Caracterizar comparativamente las medidas de políticas públicas para prohibir y regular el asbesto en América Latina y el Caribe.

**Métodos:**

Diseño cualitativo mediante el uso del método de análisis comparativo de políticas. Se consultaron fuentes documentales basados en normas nacionales vigentes y se construyeron seis categorías a partir de la codificación inductiva.

**Resultados:**

La comparación entre políticas públicas de los países de la región refleja algunos aspectos relevantes como: alcances de la prohibición, organismos multisectoriales de vigilancia y acciones sancionatorias; condiciones y excepciones para la sustitución del asbesto; actividades de prevención, monitoreo y atención en salud; disposición y manejo de residuos de asbesto y formulación de políticas o programas específicos.

**Conclusión:**

Solo un pequeño grupo de países de la región cuenta con una prohibición total del asbesto, reflejo de la situación internacional. Es importante continuar avanzando en estudios que analicen los procesos políticos y las evaluaciones de la implementación de las políticas en cada país.

## Abstract (en)

**Introduction:**

Asbestos is a mineral used in different industrial activities, but it has a significant impact on the health of the working population. Several countries have established policies to prohibit or regulate its use.

**Objective:**

Comparatively characterize public policy measures to prohibit and regulate asbestos in Latin America and the Caribbean.

**Methods:**

Qualitative design using the comparative policy analysis method. Documentary sources based on current national standards were consulted and six categories were constructed based on inductive coding.

**Results:**

The comparison between public policies of the countries of the region reflects some relevant aspects such as: scope of the prohibition, multisectoral surveillance organizations and sanctioning actions; conditions and exceptions for asbestos substitution; prevention, monitoring and health care activities; disposal and management of asbestos waste and formulation of specific policies or programs.

**Conclusions:**

Only a small group of countries in the region have a total ban on asbestos, reflecting the international situation. It is important to continue advancing in studies that analyze political processes and evaluations of policy implementation in each country *(source: MeSH, NLM).*

El asbesto o amianto comprende un grupo de minerales microcristalinos y fibrosos que se encuentran en la naturaleza 1. El amianto ha sido altamente valorado debido a propiedades como su fuerza, resistencia química y escasa conducción del calor 2, útil en la industria automotriz y textil, en la construcción de viviendas, edificios y barcos, entre otros 3.

La evidencia científica ha mostrado la relación causal del asbesto con enfermedades como el cáncer de pulmón, el mesotelioma y la asbestosis 2,3. Las fibras de asbesto son inhaladas en ambientes laborales contaminados o domésticos 4. Alrededor de 125 millones de personas a nivel mundial están expuestas en sus trabajos 5 y cada año más de 107 000 personas mueren por enfermedades relacionadas. Aunque se ha reducido en algunos países, especialmente de altos ingresos, en aquellos de medianos y bajos ingresos su uso todavía es extendido 4. El periodo de latencia entre la exposición y la enfermedad oscila entre 10 y 50 años, lo que implica que la prevalencia de diferentes patologías aumentará en los próximos 30 a 100 años, aún en países donde el asbesto está prohibido 6.

Alrededor de 60 países proscriben el asbesto, pero los principales productores todavía lo procesan y exportan a naciones con escasas o nulas regulaciones 6. La Organización Internacional del Trabajo (OIT) aprobó en 1986 el Convenio 162 que prohíbe el uso del asbesto crocidolita y exige la pulverización de todas las variedades 4. La Organización Mundial de la Salud (OMS) recomienda evitar el uso de cualquier tipo, debido a la poca evidencia para establecer el umbral de su efecto carcinogénico y a que la población expuesta a niveles muy bajos puede presentar un mayor riesgo de cáncer 4. El presente estudio tuvo como objetivo comparar las políticas de los países de la región de América Latina y el Caribe (ALC) para prohibir o regular el asbesto en sus territorios.

## MÉTODOS

Se llevó a cabo un estudio cualitativo, transversal y documental mediante el uso del análisis comparativo de políticas (ACP), un método que permite comparar decisiones de política pública. El ACP puede tener estos alcances: 1. describir similitudes y diferencias (aprender sobre políticas); 2. probar hipótesis o teorías, generar clasificaciones o tipologías, o seguir la implementación de un proceso (aprender por qué una política se desarrolla de una manera y no de otra); y 3. informar a otros países sobre un determinado aprendizaje político (aprender de la política) 7. Para este estudio se optó por la primera opción, es decir, aprender sobre las diferentes políticas para la regulación o la prohibición del asbesto en países de ALC. Adicionalmente, es un análisis orientado por casos 7, aplicable debido al número pequeño de países donde cada un constituye un caso para el estudio. Se analizó la información documental de decisiones de política como leyes, reglamentos, resoluciones u otras normas generadas por las instituciones públicas del Estado a nivel ejecutivo o legislativo.

Se buscaron y consultaron las políticas a través de las páginas web de las autoridades en salud en los países de ALC. En caso de no encontrar información por este medio, se empleó el motor de búsqueda Google, usando términos como "legislación", "políticas", "asbesto", más el nombre del país. Los criterios de inclusión fueron: a) países de ALC de habla hispana y portuguesa; b) políticas vigentes al momento del estudio, es decir, hasta 2022; c) políticas aprobadas por una autoridad pública. Aquellos documentos que no cumplían con estos criterios fueron excluidos.

Se realizó la lectura completa de los contenidos de cada política en los países de los que se obtuvo información. Se llevó a cabo la selección de fragmentos textuales con su codificación correspondiente. Surgieron inicialmente 12 códigos, los cuales fueron registrados en Microsoft Excel ®, a través de una matriz que registró la fuente, el fragmento textual y el código. Luego, se procedió a agrupar estos códigos, con lo que se generaron seis categorías inductivas, conforme al análisis temático cualitativo (Tabla 1).

Definición

**Tabla 1:** Categorías inductivas y su definición

Categoria	Definición
Alcances de la política	Las políticas nacionales prohíben total o parcialmente el asbesto
Responsabilidades sectoriales	Reconocimiento de las responsabilidades del sector salud y otros sectores sobre la regulación o prohibición
Sustitución y excepciones para el uso del asbesto	Medidas para sustituir o mantener excepciones en el uso del asbesto
Prevención, monitoreo y atención en salud	Acciones para prevenir los riesgos por la exposición, así como el seguimiento de las condiciones de salud de grupos en riesgo y atender a las personas enfermas debido al asbesto
Disposición y manejo	Estrategias para disponer o manejar adecuadamente los residuos o desechos con asbesto
Formulación de políticas y programas específicos.	Las políticas configuran otras decisiones específicas como parte de su implementación

## RESULTADOS

### Alcance de la política

De los 19 países hispanoportugueses de ALC, siete no cuentan con regulaciones sobre el asbesto: El Salvador, Guatemala, Nicaragua Panamá, Paraguay, República Dominicana, Bolivia y Venezuela. Sobre los 12 restantes, solo cinco de ellos tienen prohibiciones totales: Argentina, Colombia, Chile, Honduras y Uruguay, y los demás solo tienen medidas de regulación (Tabla 2). Es importante tener en cuenta que la ratificación al Convenio 162 OIT no implica la regulación del asbesto en los países que lo suscriben. Por ejemplo, Argentina no ha suscrito el Convenio y ha prohibido el asbesto; en cambio, Guatemala sí lo hizo, pero carece de normatividad para regularlo o prohibirlo (Tabla 2). En el caso de Colombia, la norma señala que "se prohíbe explotar, producir, comercializar, importar, distribuir o exportar cualquier variedad de asbesto y de los productos con él elaborados en el territorio nacional" 8; de forma mucho más breve, en Uruguay la norma declara así: "Prohíbase, la fabricación, la introducción al territorio nacional bajo cualquier forma y la comercialización de productos que contengan amianto" 9.

**Tabla 2:** Países de ALC, alcance de la política y vinculación al Convenio 162 OIT

País	Alcance	Convenio 162 OIT	Año
Argentina	Prohibición	No	2000
Colombia	Prohibición	Sí	2019
Costa Rica	Regulación	No	1996
Cuba	Regulación	No	2021
Chile	Prohibición	Sí	2000
Ecuador	Regulación	Sí	2000
Honduras	Prohibición	No	2004
México	Regulación	No	1994
Panamá	Regulación	No	1999
Perú	Regulación	No	2011
Uruguay	Prohibición	Sí	2002

Solo Perú y Colombia cuentan con normas expresadas en leyes aprobadas en sus respectivos congresos. En los demás países se encuentran decisiones de políticas que se manifiestan a través de resoluciones, decretos u acuerdos generados por autoridades públicas de la rama ejecutiva del Estado. Y de estos, varios provienen de las autoridades en salud o trabajo (Tabla 3).

**Tabla 3:** Normatividad, autoridades y países de ALC

País	Tipo de norma	Institución qué expide	Titulo
Argentina	Resolución	Ministerio de Salud	Resolución 845 de 2000: Prohíbase la producción, importación, comercialización y uso de fibras de asbesto variedad anfiboles y productos que la contengan 11
Brasil	Declaración de inconstitucionalidad	Supremo Tribunal Federal	Declaración de inconstitucionalidad de la Ley 9.055 de 1995 sobre el uso regulado del amianto variedad crisotilo por violar los deberes y derechos constitucionales de protección a la salud y el medio ambiente
Colombia	Ley	Congreso	Ley 1968 de 2019 "Por la cual se prohíbe el uso del asbesto en el territorio nacional y establecen garantías de protección a la salud de los colombianos" 8
Costa Rica	Decreto	Presidencia	Decreto 25056 de 1996 "Reglamento de uso controlado del asbesto y productos que lo contengan" 10
Cuba	Resolución	Ministerio de Justicia	Resolución 253/2021 "Reglamento para el manejo de los productos químicos peligrosos de uso industrial, de consumo de la población y de los desechos químicos peligrosos" 12
Chile	Decreto	Ministerio de Salud	Decreto 656 de 2000 Prohíbe uso del asbesto en productos que indica el Ministerio de salud 13*
Ecuador	Acuerdo	Ministerio de Trabajo	Acuerdo 0100 de 2000 Reglamento de seguridad para el uso del amianto 14
Honduras	Decreto	Ministerio de Salud	Decreto de Acuerdo Ejecutivo 032 de 2004 15
México	Regulación técnica	Secretaría de Salud	Norma Oficial Mexicana NOM-125-SSA1-1994 "Que establece los requisitos sanitarios para el proceso y uso del asbesto" (Actualizado NOM-125-SSA-2016) 16
Panamá	Resolución	Ministerio de Salud	Resolución No 50 de 1999 Manejo, almacenamiento y transporte de Asbesto en la República de Panamá 17
Perú	Ley	Congreso	Ley 29662 de 2011 que prohíbe el asbesto anfíboles y regula el uso del asbesto crisotilo 18
Uruguay	Decreto	Presidencia	Decreto N.o 154/002 Prohibición de la comercialización de productos que contengan amianto o asbesto 9

### Responsabilidades sectoriales

Algunos países asignan la responsabilidad de formular políticas, vigilar o sancionar las acciones que las incumplan en instituciones públicas ya existentes o en otras nuevas. En Argentina, la Secretaría de Industria y Comercio, la Administración Nacional de Aduanas, la Secretaría de Desarrollo Sustentable y Política Ambiental y la Superintendencia de Riesgos de Trabajo son entidades de diferentes sectores a las que se les asignan responsabilidades de acuerdo con sus competencias para la regulación en el plano comercial, ambiental y laboral 11.

Por su parte, en Costa Rica se señala que "El Departamento de Sustancias Tóxicas y Medicina del Trabajo, la Contraloría Ambiental del Ministerio del Ambiente y Energía, la Oficina Nacional de Normas y Unidades de Medida del Ministerio de Economía, Industria y Comercio serán responsables de la aplicación del presente Reglamento" 10, lo que indica las responsabilidades multi-sectoriales para la implementación de esta política. Además, el Ministerio de Salud tiene la función de asegurar que las personas y las instituciones protejan la salud de los trabajadores al eliminar o reducir cualquier riesgo por la exposición al asbesto 10.

En Perú y Colombia se crearon organismos intersectoriales. En el primero se encuentra la Comisión Técnica Intersectorial, responsable de velar por el cumplimiento de las disposiciones normativas; esta comisión tiene la capacidad de generar normas complementarias e imponer sanciones 18. En Colombia, se creó la Comisión Nacional para la Sustitución del Asbesto, integrada por autoridades de diferentes sectores y de la sociedad civil, y que debe supervisar el cumplimiento de las disposiciones de la ley conforme a los plazos establecidos 8.

### Sustitución y excepciones

En Argentina, se afirma que "se autorizará la comercialización y uso de productos con Asbesto para los cuales se acredite fehacientemente la imposibilidad de reemplazo o la inexistencia en el mercado, durante un plazo no mayor de un 1 año, cumplido el cual podrá ser renovada de persistir las condiciones que justificaron la autorización inicial" 11. Aquí, la sustitución está condicionada a la posibilidad de reemplazar este elemento, por lo tanto, si no hay sustitutos, puede continuarse su uso.

En Chile se ha prohibido totalmente el asbesto, pero se puede autorizar su uso en la fabricación de productos que no sean aquellos correspondientes a la construcción, con la condición de que no exista factibilidad técnica o económica para reemplazarlo por otro material 13. Esta excepcionalidad también es reconocida en otros países como Perú 18, Ecuador 14 y Costa Rica 10. En el caso colombiano la norma no establece excepciones.

### Prevención, monitoreo y atención en salud

Pocas políticas establecen acciones de prevención del riesgo, monitoreo de las condiciones de salud y prestación de servicios para vigilar su exposición e identificar impactos. En Costa Rica se exige responsabilidades a las empresas e industrias que producen materiales con asbesto, como es "difundir la información y promover la educación respecto a los riesgos que entraña para la salud la exposición al asbesto, así como los métodos de prevención y control al respecto" 10. Así mismo, su reglamento solicita medidas de protección a los trabajadores como el uso de mascarillas, uso adecuado de ropa de trabajo y exámenes periódicos, entre otras 10.

En México, los establecimientos que usen asbesto deben tener un control directo del ambiente de trabajo, midiendo los niveles de concentración, así como la periodicidad en los exámenes respiratorios de los trabajadores; por ejemplo, "En el caso de que los niveles de concentración ambiental rebasen los límites permitidos y/o se encuentren signos o síntomas clínicos y la espirometría reporte pérdida de la capacidad funcional del 31 al 51% se considerará que el trabajador está trabajando en un riesgo elevado de exposición, se le hará un seguimiento anual y un manejo médico especializado para que el daño no progrese" 16. En Colombia se creó la Ruta para la Atención Integral de las Personas Expuestas al Asbesto, que comprende no solo el suministro de información y orientación sino también medidas de atención en salud, incluidos exámenes de diagnóstico y el tratamiento 8.

## Disposición y manejo del asbesto

En la construcción, demolición o desmantelamiento de estructuras, en Chile se exige de la autorización de la autoridad sanitaria 13; en Costa Rica no se requiere autorización, pero se solicita el uso de métodos que impidan la liberación de partículas de asbesto al ambiente 10. En este país también se requiere el uso de etiquetas con un fin específico: "Será responsabilidad de los proveedores de productos que contengan asbesto, rotular los embalajes y, cuando ello sea necesario, los productos, con información sobre el riesgo y medidas de prevención en su uso y manejo de los mismos, en idioma español y de una manera fácilmente comprensible para los trabajadores y los usuarios interesados" 10.

La medida de la rotulación también es compartida por la normatividad panameña, la cual plantea: "Todos los recipientes que contengan asbesto deberán estar provistos de etiquetas colocadas en lugares visibles que identifiquen el producto y adviertan claramente de los riesgos de asbestosis y cáncer que presenta la manipulación continuada y poco cuidadosa del asbesto" 17. Así, las etiquetas cumplen la función de informar al trabajador sobre el producto que está manipulando y los riesgos que tiene su uso inadecuado.

### Formulación de políticas, planes y programas

Sólo Colombia y Perú incorporan otras políticas para abordar algún aspecto complementario alrededor del asbesto.

En el primero, la ley propone la política pública de sustitución de asbesto y el plan de adaptación laboral y reconversión productiva, dirigidos a empresas y a trabajadores respectivamente y que debe existir cinco años después de la aprobación de la norma, es decir para el año 2024 8.

En Perú, el Plan Nacional de Prevención y Control de las Enfermedades Relacionadas con el Asbesto busca prevenir y enfrentar los problemas de salud generados a los trabajadores y sus familias y el entorno social alrededor de las industrias. La formulación de este plan se encuentra a cargo de la Comisión Técnica Multisectorial 18.

## DISCUSIÓN

Este estudio tuvo como objetivo analizar comparativamente las políticas que regulan o prohíben el asbesto en países de ALC. Los resultados indican que a pesar de las recomendaciones de la OMS para que las naciones prohíban totalmente el asbesto, solamente cinco cuentan con disposiciones que en tal sentido: Argentina (2000), Chile (2000), Uruguay (2002), Honduras (2004) y Colombia (2019). Las demás, o bien siguen las orientaciones establecidas por el Convenio 162 de la OIT o no cuentan con un lineamiento regulatorio particular.

En Brasil, solo 17 de los 27 estados tienen leyes que prohíben el amianto. El Supremo Tribunal Federal de Brasil declaró en 2017 la inconstitucionalidad de la Ley 9.055 de 1995 que permitía la utilización controlada del asbesto variedad crisotilo; los efectos de esta decisión fueron vinculantes desde 2019, con el fin de que los estados adaptaran sus aparatos productivos, especialmente aquellos sin leyes de prohibición 19.

Las decisiones normativas solo son en algunos casos fueron leyes emitidas desde los congresos de los países, como en el caso de Perú y Colombia, en el marco de un proceso complejo construcción de consensos requeridos para su aprobación. En otros casos, la prohibición se estableció mediante decisiones de la rama ejecutiva en cabeza de la Presidencia o de algunos de los ministerios como en Argentina, Honduras o Uruguay.

De acuerdo Hernández et al. 20, las legislaciones en ALC han carecido de guías o mecanismos para el registro o la identificación de las personas que están o han sido expuestos en el pasado al asbesto. Así mismo, sugieren tres aspectos por mejorar en el marco normativo: 1) participación de víctimas y trabajadores en comisiones nacionales para la sustitución del asbesto; 2) campañas educativas para informar a las personas sobre la remoción de elementos que contengan asbesto, ya que una inadecuada manipulación aumenta el riesgo de exposición; y 3) periodos de seguimiento de 30 años o más en las condiciones de salud de los trabajadores expuestos al asbesto 20.

Los efectos de las decisiones políticas sobre el asbesto son de largo plazo. En Corea del Sur la prohibición comenzó en el año 2007, se hizo efectiva en 2009, más de 15 años después del primer caso de mesotelioma pleural maligno relacionado con la exposición ocupacional 21. En Hong Kong, las enfermedades relacionadas con el asbesto se presentaron desde los años noventa, pero solo hasta 2014 se contó con la legislación que prohibió totalmente este mineral 22. En Japón, solo se logró la prohibición en 2004, resultado de procesos políticos intensos que fueron favorables hacia la salud pública 23.

Glass 24 muestra cómo el interjuego de diferentes actores, incluyendo a los enfermos, sus familias, grupos de apoyo, sindicatos y sectores políticos, logró la prohibición nacional del asbesto en 2016, luego de décadas de lucha que inició en los años sesenta del siglo pasado 24. En Tailandia, se prohibió el asbesto crisotilo en 2010. El proceso de formulación de la política alcanzó importantes acuerdos intersectoriales; sin embargo, en la implementación ha habido dificultades debido al conflicto persistente entre sectores a favor de la prohibición, salud y ambiente, y sectores en contra como la industria, lo que muestra el insuficiente consenso y la resistencia alrededor de la política 25.

Dinamarca fue el primer país europeo que prohibió el asbesto, en 1972, luego Italia en 1992, España en 2001 y, finalmente todo el continente en 2005. Como sucede con otros países, a pesar de estas decisiones políticas tomadas con varios años de antelación, y debido al amplio periodo de latencia, aún no es posible evaluar los efectos de las medidas en la salud poblacional. Las exposiciones de los años setenta, ochenta y noventa, seguramente se reflejarán en la actualidad o en los próximos años con un mayor número de enfermedades 26. Además, otro reto significativo es la eliminación segura del amianto en los materiales existentes que se encuentran en edificios, trenes, máquinas, etc., lo que lleva a la configuración de otras políticas que avancen en este sentido 26.

Como conclusión, la mayoría de los países de la región han establecido estas políticas con alcances regulatorios, mientras que solo unos pocos han logrado prohibir totalmente su uso. Son indispensables estudios futuros desde el análisis de políticas para comprender los procesos políticos particulares en cada país, así como el abordaje científico sobre las experiencias en la implementación de las políticas para conocer sus avances, así como las barreras que han tenido ♣
